# Comparison of Mechanical Support with Impella or Extracorporeal Life Support in Post-Cardiac Arrest Cardiogenic Shock: A Propensity Scoring Matching Analysis

**DOI:** 10.3390/jcm10163583

**Published:** 2021-08-14

**Authors:** Styliani Syntila, Georgios Chatzis, Birgit Markus, Holger Ahrens, Christian Waechter, Ulrich Luesebrink, Dimitar Divchev, Harald Schuett, Panagiota-Eleni Tsalouchidou, Andreas Jerrentrup, Mariana Parahuleva, Bernhard Schieffer, Konstantinos Karatolios

**Affiliations:** 1 Department of Cardiology, Angiology and Intensive Care, Philipps University Marburg, 35043 Marburg, Germany; syntila@med.uni-marburg.de (S.S.); birgit.markus@med.uni-marburg.de (B.M.); holger.ahrens@med.uni-marburg.de (H.A.); christian.waechter@med.uni-marburg.de (C.W.); ulrich.luesebrink@med.uni-marburg.de (U.L.); dimitar.divchev@med.uni-marburg.de (D.D.); harald.schuett@med.uni-marburg.de (H.S.); parahuleva@med.uni-marburg.de (M.P.); bernhard.schieffer@med.uni-marburg.de (B.S.); konstantinos.karatolios@staff.uni-marburg.de (K.K.); 2 Department of Neurology, Stroke and Epilepsy Center, Philipps University Marburg, 35043 Marburg, Germany; pania.tsalouchidou@gmail.com; 3 Department of Emergency Medicine, Philipps University Marburg, 35043 Marburg, Germany; Andreas.Jerrentrup@uk-gm.de

**Keywords:** out of hospital cardiac arrest, post-cardiac arrest cardiogenic shock, mechanical circulatory support, Impella, ECLS

## Abstract

Our aim was to compare the outcomes of Impella with extracorporeal life support (ECLS) in patients with post-cardiac arrest cardiogenic shock (CS) complicating acute myocardial infarction (AMI). This was a retrospective study of patients resuscitated from out of hospital cardiac arrest (OHCA) with post-cardiac arrest CS following AMI (May 2015 to May 2020). Patients were supported either with Impella 2.5/CP or ECLS. Outcomes were compared using propensity score-matched analysis to account for differences in baseline characteristics between groups. 159 patients were included (Impella, *n* = 105; ECLS, *n* = 54). Hospital and 12-month survival rates were comparable in the Impella and the ECLS groups (*p* = 0.16 and *p* = 0.3, respectively). After adjustment for baseline differences, both groups demonstrated comparable hospital and 12-month survival (*p* = 0.36 and *p* = 0.64, respectively). Impella patients had a significantly greater left ventricle ejection-fraction (LVEF) improvement at 96 h (*p* < 0.01 vs. *p* = 0.44 in ECLS) and significantly fewer device-associated complications than ECLS patients (15.2% versus 35.2%, *p* < 0.01 for relevant access site bleeding, 7.6% versus 20.4%, *p* = 0.04 for limb ischemia needing intervention). In subgroup analyses, Impella was associated with better survival in patients with lower-risk features (lactate < 8.6 mmol/L, time from collapse to return of spontaneous circulation < 28 min, vasoactive score < 46 and Horowitz index > 182). In conclusion, the use of Impella 2.5/CP or ECLS in post-cardiac arrest CS after AMI was associated with comparable adjusted hospital and 12-month survival. Impella patients had a greater LVEF improvement than ECLS patients. Device-related access-site complications occurred more frequently in patients with ECLS than Impella support.

## 1. Introduction

Out of hospital cardiac arrest (OHCA) is a major public health problem and a leading cause of death in industrialized nations [1]. The poor survival rates of OHCA patients highlight the need to seek interventions regarding each step in the management of these patients to improve outcomes. Obviously, cardiac issues are central, as coronary artery disease remains the predominant cause of cardiac arrest (CA), and the heart is an important therapeutic target in the post-resuscitation period. Post-cardiac arrest cardiogenic shock (CS) occurs frequently after resuscitation from CA and may lead to multi-organ failure and death, even in patients with a good neurologic prognosis [2]. Optimal management is therefore crucial to further improve survival [3]. In this regard, mechanical circulatory support (MCS) may be considered at that time in order to augment cardiac output, stabilize hemodynamics, and ensure adequate organ perfusion [2]. However, the optimal selection of the device type remains unclear and, so far, no specific guideline recommendation exists. The Impella pump and extracorporeal circulatory support (ECLS) are the most frequently used device types for temporary percutaneous MCS in this context [4,5].

There are only few retrospective studies evaluating the potential benefit of the Impella pump in the particular setting of post-cardiac arrest CS [6,7]. On the other hand, studies focusing on ECLS in patients with post-cardiac arrest CS yielded conflicting results [8,9,10]. Moreover, studies comparing Impella and ECLS in large homogenous patient populations, specifically in post-cardiac arrest CS after OHCA, are missing since investigations comparing MCS have included only heterogenous populations with and without prior cardiac arrest [11,12,13]. Therefore, the aim of this study was to analyze the outcomes of OHCA patients with post-cardiac arrest CS after AMI assisted with either Impella or ECLS.

## 2. Methods

### 2.1. Study Design

We retrospectively analyzed data from all patients resuscitated from OHCA due to AMI with post-cardiac arrest shock from May 2015 to May 2020 who had been admitted to our center and who had received either Impella (2.5 or CP) or ECLS for post-cardiac arrest CS. Patients with refractory OHCA under cardiopulmonary resuscitation, patients with OHCA due to other causes than AMI, and patients with biventricular support (e.g., Impella and ECLS) were excluded. Post-cardiac arrest shock was defined as the need for the continuous infusion of vasopressors to maintain systolic blood pressure >90 mmHg after ROSC with end organ hypoperfusion indicated by an elevated lactate level >2 mmol/L. According to our institutional practices, operators were encouraged to utilize MCS in patients with post-cardiac arrest CS. However, due to insufficient evidence for the choice of the circulatory support device type in OHCA patients, the decision to implant an Impella or ECLS was based on the operator’s discretion.

The study was approved by the local ethics committee of the Philipps University of Marburg. The need for informed consent was waived due to the retrospective nature of the study.

### 2.2. Patients’ Management

All MCS devices were implanted percutaneously in the catheterization laboratory on admission day by experienced operators. The Impella pump (Abiomed, Danvers, MA, USA) was inserted through the femoral artery and was placed retrogradely through the aortic valve into the left ventricle under fluoroscopic control. ECLS (Maquet Group) was implanted percutaneously using arterial (17F) and venous (21F for female and 23F for male) femoral cannulas with an additional antegrade femoral limb perfusion cannula. All of the patients were treated with targeted temperature management (mild hypothermia of 34 °C) for 24 h with an endovascular cooling device (Thermogard Temperature Management System, Zoll Medical Corporation, Chelmsford, MA, USA) Catecholamines were used to obtain a mean arterial pressure ≥ 65 mmHg. Circulatory support flow was adjusted to maintain mean arterial pressure ≥ 65 mmHg with the lowest possible dose of catecholamines and to cover metabolic needs as assessed by central venous oxygen saturation (≥70%) and serum lactate levels (<2.0 mmol/L). The decision to wean the circulatory support device was based on the resolution of shock and clinical assessment. Once the support of the device was gradually reduced to low levels (for Impella performance level 1 and for ECLS < 1.5 L/min) with stable mean arterial pressure ≥65 mmHg, no or low doses of catecholamines, central venous oxygen saturation ≥70%, and serum lactate levels <2.0 mmol/L the device was extracted. Hemostasis was achieved using mechanical compression (FemoStop, Abbott Cardiovascular, Germany).

### 2.3. Data Collection and Outcome Variables

Intrahospital clinical data, outcomes, and follow-up data were collected from the patients’ medical charts. Prehospital arrest data were collected with the use of a preformatted standard data collection tool. Our primary outcomes were survival to hospital discharge and survival at 12 months. Secondary endpoints were complications. Complications included device-related vascular complications (access-site bleeding requiring transfusion, limb ischemia requiring extraction of the device, or surgical or interventional repair), myocardial reinfarction, stroke, and other non-device related bleeding. Access-site bleeding requiring transfusion was defined as bleeding at the cannulation site with need for the transfusion of at least three red blood cell (RBC) units. Cerebral functional status was determined according to the Pittsburgh cerebral performance category (CPC) based on neurological assessment or from medical records and discharge summary abstracts.

### 2.4. Statistical Analysis

All data were analyzed retrospectively. Data are presented as absolute variables and percentages (%) for categorical variables and are either the median with interquartile range (IQR: 25–75th percentile) or the mean with standard deviation according to the distribution of the variables. We assessed normality using the Shapiro–Wilk test as well as Pearson tests. After testing for normal distribution, Student’s *t*-test or the Mann–Whitney test was implemented to test for differences between the various characteristics. For categorical variables, Fisher’s exact test or the chi-square test were used as appropriate. Interactions between nominal variables were measured with the lambda coefficient. Patients who were at risk were assessed with the log-rank test of the survival analysis. The variables were dichotomized according to median in overall population, when they were not linearly distributed. An initial analysis was performed in order to identify the variables that were associated significantly with outcome mortality in the overall population. A separate analysis was performed in order to identify the variables with a different distribution among the groups of devices. All of these variables are presented in Table 1. The Charlson comorbidity index (CCI), vasoactive score, pH, PaO_2_/FiO_2_ (Horowitz index), lactate, first rhythm, and time from collapse to return of spontaneous circulation (ROSC) were included in the model as being significantly associated with outcome in univariate analysis or as clinically meaningful. Although age was an independent variable of outcome, we did not include it in order to avoid overfeeding of the model since the CCI was already age-adjusted. Propensity score matching was used to balance the observed covariates in the treatment groups. In this study, the propensity score was the conditional probability for receiving ECLS for CS as a binary dependent variable under a set of measurements. CCI, vasoactive score, first rhythm, PaO_2_/FiO_2_, pH, lactate, and time from collapse to ROSC were added into a multivariable logistic regression model. The predicted probability derived from the logistic equation was used as the propensity score for each individual. We then performed a conditional logistic regression after matching on the propensity score in a 1:1 in order to identify the matched pairs. All of the analyses were considered statistically significant for *p* < 0.05. All of the analyses were two-sided. Statistical analysis was performed using SPSS 24 and Graphpad Prism 6.0.

## 3. Results

From May 2015 to May 2020, a total of 159 patients with post-cardiac arrest CS after AMI and ROSC received MCS with Impella 2.5/CP (66% or 105 patients) or ECLS (34% or 54 patients), and these patients were included in the present analysis. Baseline characteristics of the patients are presented in Table 1, and the procedural characteristics of all groups are listed in Table 2.

**Figure 1:**
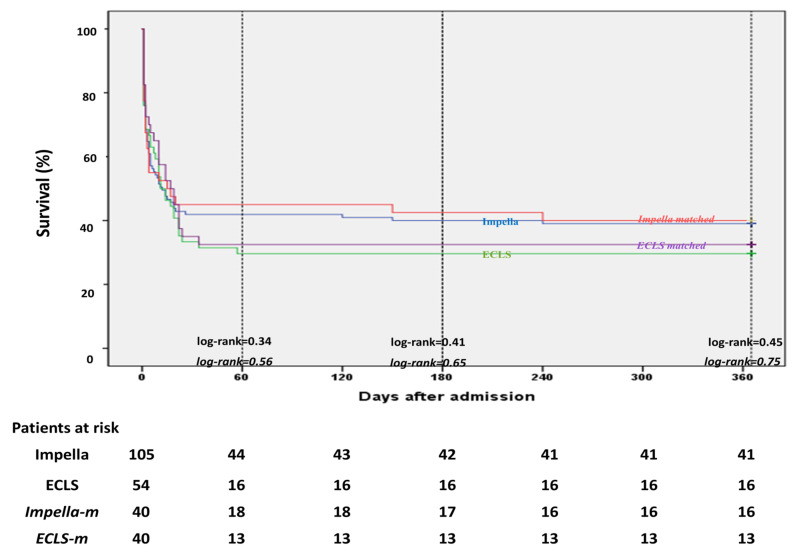
Kaplan–Meier curves demonstrating 12-month survival in Impella and ECLS patients among overall and matched cohort.

Hospital survival rates were 41.9% in the Impella group and 29.6% in the ECLS group (*p* = 0.17), whereas the overall survival rate was 37.7% (Table 3). The causes of death were anoxic brain damage in 6.9% and refractory CS/multi-organ-failure (MOF) in 55.3% of the overall cohort (Table 3). In the Impella group, refractory CS/MOF and anoxic brain damage were the causes of death in 52.4% and in 5.7% of the patients, respectively, whereas in the ECLS group, 9.3% of the patients died from brain damage and 61.1% died from refractory CS/MOF (*p* = 0.32 Impella versus ECLS for refractory CS/MOF and *p* = 0.51 Impella versus ECLS for brain death) (Table 3). At 12 months, 41 (39%) Impella and 16 (29.6%) ECLS patients were alive (*p* = 0.3) (Figure 1).

**Figure 2:**
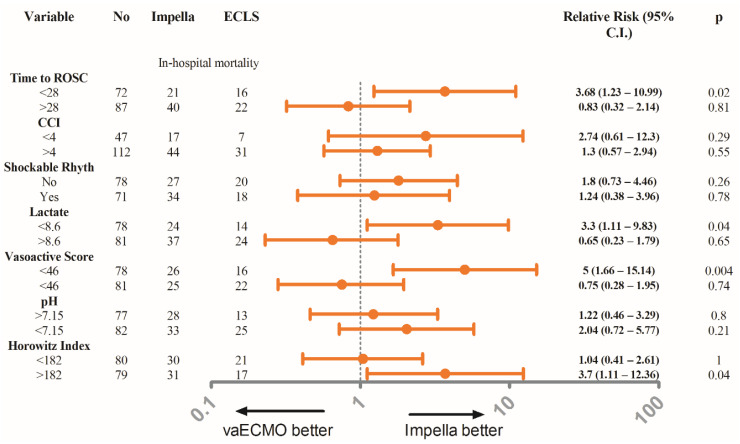
Forest plot displaying the relative risk of different tested subgroups for the primary endpoint hospital mortality in the overall cohort.

Upon admission, patients with ECLS were younger (*p* < 0.001), had a marginally longer duration of low-flow time (*p* = 0.08), significantly higher lactate levels (*p* = 0.04) and vasoactive score (*p* = 0.011), CCI (*p* = 0.02), and significantly lower pH levels (*p* = 0.04) and Horowitz Index (*p* < 0.01) compared to the patients with Impella support (Table 1). Procedural characteristics of the overall and matched cohorts are presented in Table 2. In a subgroup analysis, Impella was associated with better survival in patients with lower risk features (higher pH and Horowitz Index levels) as well in patients with lower lactate levels and a shorter time interval from collapse to ROSC. All of the variables are dichotomized on median or mean levels according to the distribution of the values (Figure 2).

Using the propensity score, 40 pairs of patients were matched. The characteristics of the propensity matched cohort were well balanced and were evenly distributed regarding the covariates (Table 1). The logistic model used to estimate the propensity score for Impella support using all of the available covariates yielded a C statistic of 0.85 (95% CI 0.74–0.91).

In the matched cohort, the hospital and 12-month survival rates were comparable in the Impella group compared to the ECLS group (hospital survival: 45% versus 32.5%, *p* = 0.36 and 12 months survival: 40% versus 32.5%, *p* = 0.64) (Table 3 and Figure 1).

**Figure 3:**
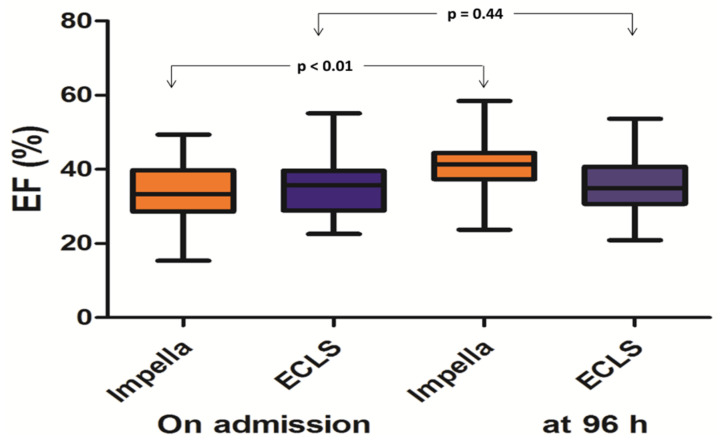
Systolic left ventricular ejection fraction on admission and at 96 h among Impella and ECLS patients demonstrating a greater functional recovery among the Impella patients.

In the Impella group, the systolic LVEF was significantly higher at 96 h compared to baseline (32.32 ± 6.89% vs. 41.33 ± 5.65% at 96 h, *p* < 0.01) and compared to ECLS, whereas in the ECLS, the systolic LVEF at baseline and 96 h were not significantly different (34.45 ± 7.21% vs. 35.38 ± 7.46% at 96 h, *p* = 0.44) (Figure 3). Systolic LVEF improved by 9.01 ± 12.44% and by 0.93 ± 8.25% in the Impella and ECLS group, respectively (*p* < 0.001).

Device-related access site complications occurred more frequently in the ECLS group than in the Impella group. Access site bleeding requiring transfusion occurred in 15.2% of the patients in the Impella group and in 35.1% of the patients in the ECLS group (*p* < 0.01) (Table 3). Vascular complications requiring intervention, such as surgery, device extraction, or percutaneous intervention occurred in 7.6% of patients in the Impella group and in 20.4% of the patients in the ECLS group (*p* = 0.04). Similarly, in the matched cohort, device related access site complications were observed more frequently in the ECLS group (Table 3). There was no significant difference regarding the incidence of non-device related bleeding, myocardial reinfarction, and stroke among the two groups (overall and matched cohort).

## 4. Discussion

Here, we describe a large single-center, propensity-matched analysis of patients with post-cardiac arrest CS after AMI supported with either Impella or ECLS. To the best of our knowledge, this is the largest single-center cohort comparing Impella with ECLS in OHCA patients with post-cardiac arrest CS following AMI. In our study, Impella and ECLS were associated with comparable hospital and 12-month survival in the overall cohort (Figure 1). However, several baseline characteristics, such as longer time to ROSC, a higher vasoactive score and lactate as well as a lower Horowitz index score and pH led to considering the ECLS group as a higher-risk population compared to the Impella group. These parameters are known to be associated with more severe post-cardiac arrest shock and worse outcome [14,15,16,17]. Therefore, since we documented these important differences, we performed propensity matching in order to improve the comparability between the two groups. In the propensity matched cohort, the survival rates at hospital discharge and 12 months were still comparable between the Impella and ECLS groups.

Given the persistently poor outcomes in resuscitated patients, interest in the role and appropriate selection of these two circulatory devices has been increasing. However, to date, very few studies have compared the characteristics and outcomes of patients treated with these two MCS devices in AMI-related CS, and no randomized data are available favoring one type of device over the other in CS, especially in the specific setting of post cardiac arrest CS [13,18,19]. In a recent study from Lemor and colleagues in patients with AMI-related CS, Impella was associated with improved clinical outcomes, fewer complications, shorter length of hospital stay, and lower hospital cost compared to those undergoing ECLS placement [20]. However, the study population was determined through ICD-10 codes using the National Inpatient Sample without any data regarding the hemodynamic profile or prior resuscitation of the patients who were included, which are major determinants of outcome in CS patients. In a previous study from our working group, treatment with either Impella 2.5/CP or ECLS was associated with better outcome in post-cardiac arrest patients in subgroup analyses [11]. However, this investigation also included patients with non-AMI related CS as well as patients with in-hospital cardiac arrest; these parameters are associated with different prognoses in post cardiac arrest CS [1,21]. Recently, Garan and colleagues prospectively compared the efficacy and outcomes of Impella and ECLS in 51 patients with AMI-related CS [13]. Their data demonstrated that the two devices were associated with similar outcomes in AMI-related CS, with survival rates higher than in our group [13]. However, in this study, only 33.3% of the patients had suffered prior cardiac arrest, only 68.6% were mechanically ventilated, and lactate levels on admission were lower, reflecting the higher mortality risk of our cohort [13]. Moreover, adjusted mortality rates in CS patients treated with ECLS or Impella were again similar in a retrospective analysis of two European registries [12]. Patients included in this analysis (*n* = 149) had high 30-day mortality rates of 70% and 83% in the Impella and ECLS groups, respectively. In this study, mortality rates were higher than in ours although only 55% of the patients had prior cardiac arrest. In another study by Karami and colleagues, Impella or ECLS support was again not associated with a difference in 30-day mortality [22]. Furthermore, even after adjustment for disease severity through the SAVE score, short- and long-term survival was not measurably different between the Impella and ECLS supported patients with CS in the retrospective analysis by Schiller and colleagues [18].

Studies focusing only on patients with post-cardiac arrest shock treated with ECLS yielded survival rates comparable to ours [8,9,10]. In particular, Ouweneel et al. [10] demonstrated a survival of 27% in a meta-analysis of ECLS use in patients with post-cardiac arrest CS due to an AMI, while De Chambrun et al. [8] reported a hospital and 12-month survival rate of 28% and 27%, and Bougouin et al. [9] observed a discharge survival rate of 25%, corroborating the high mortality of those patients treated with ECLS. In contrast, the overall survival rate was favorable when compared to other studies focusing on Impella support in post-cardiac arrest shock [6,23]. The retrospective study by Manzo-Silbermann and colleagues reported a poor survival rate at 28 days of 23% in the Impella group [6]. So far, only the randomized IMPRESS trial by Ouweneel and colleagues [23], which included almost exclusively resuscitated patients with STEMI-related CS, reported a lower mortality of 46% at 30 days and 50% at 6 months in the Impella group, compared to our results. However, we consider our Impella patients to be of greater risk, including both STEMI and NSTEMI, with a higher incidence of a non-shockable first rhythm (35.2% vs. only 9% in the IMPRESS trial), a longer duration of CPR, and higher lactate levels on admission. It has repeatedly been shown that these parameters are associated with worse prognosis in resuscitated patients [14,15,16,17,23,24].

Another important finding of our study was the higher functional recovery with a significantly greater improvement of LVEF in Impella patients compared to the ECLS group (Figure 2). Adverse effects of ECLS on the failing heart increase left ventricular wall stress are due to retrograde blood flow [25,26]. Lack of LV unloading and increased afterload may lead to ventricular distension, myocardial ischemia, and worsening pulmonary edema, especially in patients suffering from severe acute LV dysfunction caused by direct damage of the ischemic myocardium [25,27,28]. These known adverse effects of ECLS may impede myocardial recovery and increase mortality [26,29]. On the other hand, Impella unloads the LV and decreases LVEDP more than ECLS in failing hearts in animal models and reduces LV end-diastolic wall stress, increasing coronary perfusion to the infarcted region in a porcine model of ischemic heart failure [26,30]. Moreover, Impella support augments coronary flow [31]. The reduction in oxygen demand by decreasing LV wall stress and PVA combined with an increase in coronary flow may minimize the damage after an ischemic insult and may reduce myocardial infarction size [32]. These findings suggest that LV support and unloading with Impella provides a better recovery potential in acute pump failure than with ECLS, which reflects the greater LVEF improvement in the Impella group observed in our analysis.

Access site complications occurred more frequently in the ECLS group than in the Impella group. Complications with MCS devices are variably reported on in the literature. Recently, a registry including 112 patients assisted with Impella for AMI-related CS reported overall vascular complications in17% of patients, limb ischemia in 3.5% of patients, and major access-site bleeding in 9.8% of patients, which is comparable to the access-site complication rates in our study [33]. In another retrospective analysis of patients with AMI related CS treated with Impella reported a higher rate of peripheral ischemic vascular complications of 9.8% of patients despite a lower coincidence of peripheral arterial disease (PAD) at 14.2%, compared to our investigation [34]. In patients treated with ECLS cannulation-site, vascular complications occur at a higher rate, with femoral bleeding rate occurring in 32% of patients and a lower extremity ischemia rate in 16.9–20% of patients [35,36]. In particular, in the setting of post-cardiac arrest shock, access-site bleeding and limb ischemia occurred at a rate of 26–29% and 8–15%, respectively [8,9]. In our ECLS cohort, the vascular complication rate was slightly higher (access-site bleeding 35.1% and limb ischemia 20%). This could be attributed to the fact that our analysis only included patients with AMI on dual antiplatelets treatment and that concomitant PAD was present in 42% of patients.

Despite the fact that MCS is becoming an increasingly integral part of the management of refractory CS, the best strategy remains unclear, and until now, no device has been shown to provide a clear survival benefit over the other in this setting. However, CS is a dynamic entity, which ranges from light forms that only necessitate temporally lower doses of catecholamines to severe refractory forms with very high mortality rates despite high doses of catecholamines and MCS. As such and due to the lack of the randomized data, it is important for the treating physician to guide therapy and the selection of the proper MCS device according to individual patient characteristics and indices of CS. In this regard, we analyzed the effects of the MCS device according to several well-known predictors of outcome in patients with post cardiac arrest CS (Figure 3). Impella was associated with better outcome in patients with lower lactate levels, shorter time from collapse to ROSC as well as with lower vasoactive score. A possible explanation for the superiority of Impella in patients with lower risk characteristics may be the higher complication rates observed in the ECLS patients (Table 3). Another possible explanation may be the higher functional recovery with a greater LVEF improvement in Impella patients compared to in the ECLS group (Figure 2). However, in patients with severe and profound CS, this advantage could be well counterbalanced by the severity of the illness and other parameters, including neurological and non-cardiac factors. In every case, the subgroup analysis should be interpreted with caution and needs to be confirmed in larger studies.

### Limitations

This study has several limitations. First, our observations are obviously limited by the retrospective and non-randomized design of our study, and therefore, the results should be interpreted according. Second, detailed right heart catheter hemodynamic data before and after device implantation device were not available for all patients. However, in emergency situations, extensive invasive hemodynamic measurements are often not performed. Moreover, we could only retrieve adverse events and complications that were properly documented in the patients’ charts. We therefore focused on mortality outcomes, as the primary endpoints were well documented in our MCS registry. Furthermore, device-related hemolysis could not be sufficiently assessed due to the absence of routine measurements of plasma-free hemoglobin in our institution. Finally, there were no cases with Impella 5.0. However, the Impella 5.0 needs cardiothoracic support and surgical placement, meaning that it would not always be feasible in emergency settings or in centers lacking cardiothoracic departments. Given the non-randomized design of this study, the results remain preliminary, and all associations need to be evaluated in future prospective and randomized trials.

On the other hand, our study has several strengths. First, our analysis included the largest population with post-cardiac arrest shock following AMI treated with percutaneous MCS. Second, the setting was quite homogenous, exclusively including patients with post-cardiac arrest CS after AMI, avoiding interactions with other forms of CS that may have a different course or prognosis, while there were no significant differences regarding the management between the Impella and ECLS group. Third, the matched study groups were well balanced allowing for a fair comparison. Last but not least, the subgroup analysis represents another strength of our study since such information might help treating physicians to guide CS therapy more effectively, enhancing the chance of survival and reducing futile healthcare.

## 5. Conclusions

In this retrospective study, use of Impella 2.5/CP or ECLS in patients with post-cardiac arrest CS after AMI was associated with similar adjusted hospital and 12-month survival rates. In Impella patients, a greater LVEF improvement was observed compared to in ECLS patients. Device-related access site complications occurred more frequently in patients with ECLS than those with Impella support. Future prospective, randomized studies are needed to validate these results.

## Figures and Tables

**Table 1:** Demographics and baseline characteristics from all patients and matched groups.

Variable	All Patients (*n* = 159)	Impella (*n* = 105)	ECLS (*n* = 54)	*p*-Value	Impella (*n* = 40)	ECLS (*n* = 40)	*p*-Value
Age (years)	66.91 ± 11.8	67.56 ± 13.65	61.76 ± 10.38	<0.001	67.68 ± 12.1	62.05 ± 10.83	0.03
Gender (male/female)	125/34	81/24	44/10	0.68	30/10	31/9	1
BMI (kg/m^2^)	27.76 ± 4.09	27.63 ± 3.73	28 ± 4.75	0.6	28.38 ± 3.88	27.61 ± 4.59	0.42
Baseline LVEF (%)	32.32 ± 6.89	32.32 ± 6.89	34.45 ± 7.21	0.07	32.91 ± 6.74	34.89 ± 7.61	0.2
STEMI on presentation, *n* (%)	77 (48.4)	49 (46.7)	28 (51.9)	0.62	16 (40)	19 (47.5)	0.66
Medical comorbidities							
Hypertension, *n* (%)	118 (74.2)	79 (75.5)	39 (72.2)	0.7	26 (65)	30 (75)	0.46
Diabetes, *n* (%)	58 (36.4)	38 (36.2)	20 (37)	1	13 (32.5)	12 (30)	1
PAD, *n* (%)	56 (35.2)	33 (31.4)	23 (42.6)	0.22	14 (35)	15 (37.5)	1
Stroke, *n* (%)	14 (8.8)	8 (7.6)	6 (11.1)	0.56	4 (10)	5 (12.5)	1
PCI, *n* (%)	50 (31.4)	32 (30.5)	18 (33.3)	0.72	12 (30)	13 (32.5)	1
CABG, *n*(%)	20 (12.6)	12 (11.5)	8 (14.8)	0.62	5 (12.5)	6 (15)	1
Myocardial infarction, *n* (%)	58 (36.5)	36 (34.3)	22 (40.7	0.49	14 35)	16 (40)	0.82
CAD	65 (40.9)	40 (38.1)	25 (46.3)	0.39	14 (35)	18 (45)	0.5
Charlson Comorbidity Index	4 (3–6)	4 (2.5–6)	5 (4–7)	0.02	4.13 ± 2.69	4.78 ± 2.09	0.23
Cardiac arrest variables							
Witnessed arrest, *n* (%)	128 (80.5)	84 (80)	44 (81.5)	1	29 (72.5)	31 (77.5)	0.8
Bystander CPR, *n* (%)	120 (75.5)	80 (76.2)	40 (74.1)	0.85	29 (72.5)	29 (72.5)	1
First rhythm VT or VF, *n* (%)	96 (60.4)	68 (64.8)	28 (51.9)	0.13	27 (67.5)	23 (57.5)	0.49
No flow time (min)	4 (2–8)	4 (1.5–7)	5 (2–9)	0.22	4 (2–6.75)	4.5 (2.25–8.75)	0.32
Low flow time (min)	25.80 ± 14.29	24.45 ± 13.99	28.43 ± 14.64	0.08	23–75 ± 12–2	23–28 ± 11–92	0.86
Time till ROSC (min)	30.61 ± 14.87	28.9 ± 14.92	33.94 ± 14.32	0.04	28–13 ± 14–2	28–45 ± 10–62	0.91
Epinephrine during CPR, *n* (%)	152 (95.6)	98 (93.3)	54 (96.4)	0.1	39 (97.5)	40 (100)	1
Total epinephrine during CPR (mg)	5 (3–8)	4 (2–6.5)	7 (4.75–9)	0.005	4 (2–6)	6–7 ± 2–95	0.003
Catecholamines							
Dobutamine, *n* (%)	93 (58.5)	61 (58.1)	32 (59.3)	1	21 (52.5)	25 (62.5)	0.5
Dobutamine (µg/kg/min)	5.88 (4.28–7.49)	5.55 (3.7–7.27)	6.05 (5.33–8.38)	0.04	5.57 ± 2–29	6.34 ± 2–38	0.27
Norepinephrine, *n* (%)	159 (100)	105 (100)	54 (100)	1	40 (100)	40 (100)	1
Norepinephrine (µg/kg/min)	0.47 ± 0.32	0.4 ± 0.24	0.6 ± 0.41	0.002	0.44 ± 0.27	0.43 ± 0.25	0.87
Epinephrine, *n* (%)	36 (22.6)	23 (14.7)	13 (24.1)	0.84	7 (17.5)	7 (17.5)	1
Epinephrine (µg/kg/min) *	0.35 (0.17–0.67)	0.47 (0.32–0.65)	0.35 ± 0.29	0.25	0.27 (0.14–0.97)	0.47 (0.32–0.65)	0.4
Vasoactive Score **	61.30 ± 43.47	55.04 ± 42.25	73.46 ± 43.61	0.011	60.33 ± 36.34	56.32 ± 30.46	0.59
Mechanical ventilation	159 (100)	105 (100)	54 (100)	1	40 (100)	40 (100)	1
Hemodynamic variables on admission							
Heart rate (bpm)	88.77 ± 24.02	88.31 ± 22.21	89.67 ± 27.42	0.74	91.38 ± 22.76	91.35 ± 27.72	1
Systolic Blood Pressure (mmHg)	97.30 ± 22.20	98.53 ± 25.54	95.57 ± 20.41	0.63	98.53 ± 25.54	94.28 ±20.83	0.42
Diastolic Blood Pressure (mmHg)	56.06 ± 14.33	57.25 ± 15.56	53.80 ± 11.56	0.32	57.25 ± 15.56	53.68 ± 10.46	0.23
Mean blood pressure (mmHg)	69.81 ± 15.56	71.01 ± 17.66	67.98 ± 10.93	0.44	71.01 ± 17.66	67.58 ± 10.58	0.29
Blood values on admission							
Lactate (mmol/L)	9.05 ± 3.95	8.59 ± 3.93	9.01 ± 3.56	0.038	9.94 ± 3.86	8.95 ± 3.37	0.94
GFR (mL/min)	49.78 ± 20.4	49.02 ± 20.51	50.67 ± 20.43	0.67	51.73 ± 19.48	53.95 ± 22.31	0.68
Creatinine (mg/dl)	1.52 ± 0.7	1.52 ± 0.52	1.52 ± 0.87	0.97	1.4 ± 0.37	1.4 ± 0.84	0.99
Arterial pH	7.16 ± 0.16	7.18 ± 0.16	7.13 ± 0.16	0.04	7.18 ± 0.14	7.16 ± 0.15	0.43
PaO2/FiO2	194 ± 104.9	210.1 ± 115.3	162.5 ± 72.07	0.006	175.6 ± 103.3	179 ± 68.98	0.86

BMI: body mass index; LVEF: left ventricular ejection fraction; STEMI: ST-elevation myocardial infarction; PAD: peripheral artery disease; PCI: percutaneous coronary intervention; CABG: coronary artery bypass graft; CAD: coronary artery disease; GFR: glomerular filtration rate; CABG: coronary artery bypass graft; VT: ventricular tachycardia; VF: ventricular fibrillation; CPR: cardiopulmonary resuscitation; ROSC: return of spontaneous circulation. Numbers are presented as mean (± standard deviation), median (interquartile range, IQR 25th–75th percentile) or frequency (percentile). * Dose among patients receiving epinephrine on admission. ** vasoactive score = dobutamine (μg/kg/min) + 100 × epinephrine dose (μg/kg/min) +100 × norepinephrine dose (μg/kg/min).

**Table 2:** Procedural characteristics of the total group and matched cohorts.

Variable	All Patients (*n* = 159)	Impella (*n* = 105)	ECLS (*n* = 54)	*p*-Value	Impella Matched (*n* = 40)	ECLS Matched (*n* = 40)	*p*-Value
Door to MCS (min)	108.6 ± 55.79	109 ± 55.69	108.1 ± 56.47	0.93	107.4 ± 52.58	100.9 ± 53.91	0.61
Duration of support (hours)	72 (18.5–130.5)	65.5 (14–127.5)	88.5 (23.75–141.8)	0.19	48 (9.5–147)	105 (24–146.3)	0.15
Door to Balloon (min)	87.71 ± 44.95	84.96 ± 40.69	92.24 ± 51.28	0.35	85.88 ± 35.8	85.4 ± 50.72	0.96
Time from ROSC to hospital admission (min)	74.21 ± 37.57	73.61 ± 35.88	75.17 ± 40.46	0.81	73.03 ± 36.89	76.35 ± 43.85	0.73
Culprit vessel, (*n* %)				NS for all comparisons			NS for all comparisons
Left main	7 (4.4)	5 (4.8)	2 (3.7)		1(2.5)	2 (5)	
LAD	86 (54.1)	58 (55.2)	28 (51.9)		19 (47.5)	20 (50)	
LCx	30 (18.9)	19 (18.1)	11 (20.4)		9 (22.5)	8 (20)	
RCA	28 (17.6)	18 (17.1)	10 (18.5)		7 (17.5)	8 (20)	
Bypass-graft	8 (5)	5 (4.8)	3 (5.5)		4 (10)	2 (5)	
Multivessel disease *	107 (67.3)	78 (74.3)	39 (72.2)	0.85	28 (70)	21 (52.5)	0.17
Multivessel Intervention	41 (25.8)	24 (22.9)	17(31.5)	0.26	9 (22.5)	10 (25)	1
Successful PCI	156 (98.1)	103(98.1)	53 (98.1)	1	40 (100)	40 (100)	1
Contrast Agent (mL)	276.6 ± 123.7	287.5 ± 121.9	259.3 ± 125.8	0.19	292.5 ± 127.0	244.2 ± 119.1	0.11

ROSC: return of spontaneous circulation; LAD: left coronary artery, LCx: left circumflex artery, RCA: right coronary artery; PCI: percutaneous coronary intervention. Numbers are presented as mean (±standard deviation), median (interquartile range, IQR 25th–75th percentile) or frequency (percentile); NS: non significant. * >50% stenosis in non-culprit vessel.

**Table 3:** Clinical outcomes and complications.

Outcome	All Patients (*n* = 159)	Impella (*n* = 105)	ECLS (*n* = 54)	*p*-Value	Impella Matched (*n* = 40)	ECLS Matched (*n* = 40)	*p*-Value
Survival to hospital discharge, *n* (%)	60 (37.7)	44 (41.9)	16 (29.6)	0.17	18 (45)	13 (32.5)	0.36
• CPC 1–2, *n* (%)	41 (68.3)	30 (68.1)	11 (68.8)	1	14 (77.8)	11 (84.6)	1
• CPC 3–4, *n* (%)	19 (31.7)	14 (31.9)	5 (31.2)	1	4 (22.2)	2 (15.4)	1
Survival at 12 months, *n* (%)	57 (35.8)	41 (39)	16 (29.9)	0.3	16 (40)	13 (32.5)	0.64
Mortality to discharge, *n* (%)	99 (62.3)	61 (58.1)	38 (71.4)	0.83	22 (55)	27 (67.5)	0.36
Causes of death							
• Cardiogenic shock/MOF	88 (88.9)	55 (90.1)	33 (86.4)	0.74	20 (90.1)	25 (92.6)	1
• Brain death	11 (11.1)	6 (9.9)	5 (13.6)		2 (9.1)	2 (7.4)	
Complications							
Access site bleeding requiring transfusion, *n* (%)	35 (22)	16 (15.2)	19 (35.1)	<0.01	4 (10)	13 (32.5)	<0.01
Limb ischemia requiring intervention, *n* (%)	19 (14.7)	8 (7.6)	11 (20.4)	0.04	1 (2.5)	8 (20)	0.03
Myocardial Reinfarction, *n* (%)	1 (0.6)	0 (0)	1 (1.9)	1	0 (0)	0 (0)	1
Pericardial effusion needing paracentesis, *n* (%)	2 (1.3)	1 (1)	1 (1.9)	1	0 (0)	0 (0)	1
Stroke, *n* (%)	3 (1.9)	1 (1)	2 (3.7)	0.29	0 (0)	0 (0)	1
Non-device related bleeding, *n* (%)	8 (5)	5 (4.8)	3 (5.6)	1	2 (5)	2 (5)	1

CPC: cerebral performance category; MOF: multiorgan failure.

## Data Availability

The data presented in this study are available on request from the corresponding author. The data are not publicly available due to ethical restrictions.
